# Functional Connectivity within the Frontal–Striatal Network Differentiates Checkers from Washers of Obsessive-Compulsive Disorder

**DOI:** 10.3390/brainsci12080998

**Published:** 2022-07-28

**Authors:** Jianping Yu, Minyao Xie, Shasha Song, Ping Zhou, Fangzheng Yuan, Mengyuan Ouyang, Chun Wang, Na Liu, Ning Zhang

**Affiliations:** 1 The Affiliated Brain Hospital of Nanjing Medical University, 264 Guangzhou Road, Nanjing 210029, China; 18706748847@163.com (J.Y.); xmm139131@163.com (M.X.); 13227842896@163.com (S.S.); oymy1998@163.com (M.O.); fm51109@163.com (C.W.); zn6360@126.com (N.Z.); 2 Department of Medical Psychology, The Affiliated Brain Hospital of Nanjing Medical University, 264 Guangzhou Road, Nanjing 210029, China; zp9207@126.com; 3 School of Psychology, Nanjing Normal University, 122 Ninghai Road, Nanjing 210024, China; fulafulashantu@163.com

**Keywords:** obsessive-compulsive disorder, symptom subtypes, resting-state functional magnetic resonance imaging, ROC curve

## Abstract

Background: Obsessive-compulsive disorder (OCD) is a psychiatric disorder with high clinical heterogeneity manifested by the presence of obsessions and/or compulsions. The classification of the symptom dimensional subtypes is helpful for further exploration of the pathophysiological mechanisms underlying the clinical heterogeneity of OCD. Washing and checking symptoms are the two major symptom subtypes in OCD, but the neural mechanisms of the different types of symptoms are not yet clearly understood. The purpose of this study was to compare regional and network functional alterations between washing and checking OCD based on resting-state functional magnetic resonance imaging (rs-fMRI). Methods: In total, 90 subjects were included, including 15 patients in the washing group, 30 patients in the checking group, and 45 healthy controls (HCs). Regional homogeneity (ReHo) was used to compare the differences in regional spontaneous neural activity among the three groups, and local indicators were analyzed by receiver operating characteristic (ROC) curves as imaging markers for the prediction of the clinical subtypes of OCD. Furthermore, differently activated local brain areas, as regions of interest (ROIs), were used to explore differences in altered brain functioning between washing and checking OCD symptoms based on a functional connectivity (FC) analysis. Results: Extensive abnormalities in spontaneous brain activity involving frontal, temporal, and occipital regions were observed in the patients compared to the HCs. The differences in local brain functioning between checking and washing OCD were mainly concentrated in the bilateral middle frontal gyrus, right supramarginal gyrus, right angular gyrus, and right inferior occipital gyrus. The ROC curve analysis revealed that the hyperactivation right middle frontal gyrus had a better discriminatory value for checking and washing OCD. Furthermore, the seed-based FC analysis revealed higher FC between the left medial superior frontal gyrus and right caudate nucleus compared to that in the healthy controls. Conclusions: These findings suggest that extensive local differences exist in intrinsic spontaneous activity among the checking group, washing group, and HCs. The neural basis of checking OCD may be related to dysfunction in the frontal–striatal network, which distinguishes OCD from washing OCD.

## 1. Introduction

Obsessive-compulsive disorder (OCD) is a heterogeneous mental disorder with a lifetime prevalence of approximately 2–3% worldwide [1,2]. OCD is characterized by the presence of obsessions and/or compulsions. However, OCD patients often manifest different clinical symptoms. Inconsistent findings of previous genetic and neurobiological studies have been attributed to the heterogeneous nature of OCD phenotypes [3]. Therefore, in order to better understand and treat OCD, the subtypes of OCD have been divided to obtain clinical subgroups with better consistency. For the present study, the symptom dimensions, dysfunctional beliefs, comorbidity, age of onset, response to treatment, genetic vulnerability, and other clinical characteristics were used as an indicator of subgroup classification. The method used to classify clinical subgroups based on the symptom dimension is the most widely used. Based on a factor analysis, the following five consistent OCD subtypes were identified: contamination/washing, symmetry/ordering, doubt/aggression/checking, somatic/religious/sexual mental compulsions, and hoarding [4]. Distinguishing homogeneous subtypes of symptom to construct the multidimensional OCD model may be facilitate to the etiology of OCD. 

The most common OCD symptoms are the washing and checking dimensions, which are also the most widely investigated symptoms [5]. The checking dimension involves intrusive thoughts related to pathological doubt and harm and repeated checking behaviors to reduce uncertainty, prevent harm, or diminish feelings that things are not just right [6]. In general, the broader definition of checking compulsion is behavior characterized by repeatedly seeking assurance from others or asking for much information support to repeatedly verify ideas before making a decision [7]. OCD patients with checking symptoms tend to present excessive uncertainty and pathological doubt in symptom-related events, which may be related to a dysfunction in logical reasoning [8,9]. Distrust of sensory information/self-doubt is also an important feature of OCD individuals with checking symptoms [10]. OCD patients with washing symptoms are generally characterized by the presence of contamination-related obsessions and cleaning behaviors due to the fear of contamination. Some studies have also noted that disgust plays an important role in the symptomatology of OCD, especially in relation to OCD patients with washing themes [11]. Disgust can trigger aversion behavior and the tendency to avoid an uncomfortable stimulus. More importantly, there is an assessment process of the potential harm of objects before the appearance of an aversive response, and OCD patients with washing symptoms may have dysfunction in this process, leading to the overestimation of the threat of contaminants and the consequences of exposure [12]. Such an inappropriate cognitive assessment may prompt OCD patients to perform avoidance or compulsion (e.g., excessive cleaning behavior) to temporarily alleviate anxiety and further negatively reinforce compulsion [13]. Therefore, disgust may be related to the development and maintenance of OCD [14].

The pathophysiology of the different symptom dimensions of OCD is not fully understood. Previous neuroimaging studies have found that checking and washing OCD depend on distinct neurobiological substrates. The dorsal prefrontal cortex, anterior cingulate cortex, putamen, and amygdala are hyperactivated in association with washing OCD [15,16,17]. However, hyperactivation in the inferior frontal cortex, insula, parahippocampal regions, and right caudate is associated with washing OCD [18,19,20,21,22,23]. In general, checking OCD may be closely related to the frontal cortical network, whereas washing OCD is related to the frontal limbic network [24]. However, as the neural mechanisms of OCD have been studied in depth, studies have increasingly reported additional brain regions associated with cleaning and checking dimensional symptoms. A recent meta-analysis reported that the right orbitofrontal cortex, bilateral anterior cingulate cortex, left middle occipital gyrus, and right caudate body were more highly activated in washing OCD patients than in controls [25]. Another study also reported that the activity and connectivity of motor cortices were elevated in checking OCD [26]. The inconsistent findings across previous studies exploring the neural mechanisms of different symptom dimensions in OCD patients may be due to the use of the methods of task provocation, inconsistent criteria for classifying symptom subgroups, task-specific relevant brain area activation, and small sample sizes. Further research is still needed to construct a multidimensional model of OCD based on neuroimaging approaches to explain the underlying symptom subtypes. 

Resting-state functional magnetic resonance imaging (rs-fMRI) can reflect deficits in the intrinsic or disease-related neural activity of patients with psychiatric disorders and may be useful in revealing the pathology of neuropsychiatric disorders [27]. Regional homogeneity (ReHo) reflects the regional synchronization of spontaneous brain activity [28]. Functional connectivity (FC), an important indicator to of global properties of brain function, can reveal the collaborative relationship of spontaneous neuronal activity in different brain regions [29]. It has been suggested that functional brain activity can be explored in terms of functional segmentation and functional integration based on a single rs-fMRI signal to explore dysfunction of brain regions and by tracing the correlation between all rs-fMRI signals to explore the multiple brain regions synergistic effects [30]. Moreover, the local and global functional characteristics of the resting-state brain function are highly closely linked, such that regional brain dysfunction found by abnormal ReHo may cause abnormalities in FC between regional brain regions [31,32]. Therefore, the use of these rs-fMRI measures to obtain information on functional brain activity from two dimensions of local brain activity and functional connectivity may reveal the pathophysiological mechanisms of psychiatric disorders in a more comprehensive and in-depth manner.

In this study, we used regional homogeneity (ReHo) to compare the differences in local spontaneous neural activity among checking OCD patients, washing OCD patients, and healthy controls based on resting-state functional magnetic resonance imaging (rs-fMRI), and receiver operating characteristic (ROC) curves were used to analyze the sensitivity and specificity of local indicators as imaging markers for the prediction of OCD clinical subtypes and disease targets. The ROIs of abnormal local spontaneous brain activity were identified. A seed-based functional connectivity (seed-based FC) analysis was used to explore the differences in functional alterations between checking and washing OCD. We attempt to explain the possible neural mechanisms of different symptomatic subtypes of OCD in multiple dimensions from local functional properties to global functional network properties and provide a basis for understanding the disease mechanisms and clinical diagnosis. In view of previous findings, we hypothesized that checkers and washers have distinct neural mechanisms regulated by different brain networks as follows: the checking subtype is regulated by the frontal subcortical network, and the washing subtype is regulated by the frontal limbic network.

## 2. Methods

### 2.1. Participants

The OCD patients (n = 45) were clinical outpatients recruited between July 2020 and February 2022 from the OCD clinic, Department of Medical Psychology of Nanjing Brain Hospital, affiliated with Nanjing Medical University. We recruited patients using the following inclusion criteria: (1) primary diagnosis of OCD by an experienced psychiatrist according to the Diagnostic and Statistical Manual of Mental Disorders (Fifth edition, DSM−5), (2) confirmation of OCD diagnosis using the Mini-International Neuropsychiatric Interview (MINI), (3) score ≥ 16 on the Yale–Brown Obsessive-Compulsive Scale (Y-BOCS), (4) an age of 13–55 years, and (5) junior high school education or above. The exclusion criteria were as follows: (1) current comorbid schizophrenia, (2) neurological disorders and severe somatic disorders, (3) pregnancy and/or breastfeeding, (4) severe suicidal self-injurious behavior or at risk of suicide attempts, and (5) inability to complete MRI.

Healthy controls (HCs, n = 45) matched for gender, age, and education were recruited through internet advertisements and posters in the community. We used the following inclusion criteria for the HCs: (1) an age of 13–55 years, (2) people who volunteered to participate in the study, and (3) junior high school education or above. In addition, MINI was used to exclude psychiatric disorders in HCs. The exclusion criteria were as follows: (1) neurological disorders and severe physical illnesses, (2) a family history of any psychiatric disorders and neuropsychiatric disorders, (3) a history of taking psychotropic drugs or psychoactive substances within the past three months, (4) pregnancy and/or breastfeeding, (5) severe suicidal self-injurious behavior or at risk of suicide attempts, and (6) inability to complete MRI. This study (No. 2020-KY208.01) was approved by the Research Ethics Committee at Nanjing Brain Hospital, Nanjing Medical University, and informed consent was obtained from each subject.

### 2.2. Clinical Measures and Quality Control

After diagnosis, a self-reported demographic questionnaire was used to collect the general information of all participants, including their age, gender, education level, history of smoking and drinking, family history, history of physical illness, duration of illness, history of psychotropic medication, and history of psychotherapy. Furthermore, the severity of OC symptoms and the symptom profile of each patient were clinically evaluated by using Y-BOCS [33]. The Obsessive-Compulsive Inventory-Revised (OCI-R) was used by the patient themselves to identify the severity of the different symptom dimensions of OCD [34]. The severity of anxiety and depression symptoms was assessed by the Beck Anxiety Inventory (BAI) and Beck Depression Inventory (BDI) of each patient, respectively [35]. Previous literature showed that the above scales have great validity and reliability [36,37,38,39].

To establish the composition of the assessment team, a psychiatry senior title clinician served as the supervisor of the team who regularly trained and consistently evaluated the team members, followed by the completion of other relevant assessment scales by residents who were uniformly trained and passed a consistent evaluation. 

### 2.3. Clinical Subtype Classification

The Yale–Brown Obsessive-Compulsive Scale Checklist (YBOCS-SC) and the method of symptom typing for OCD by Murayama [16] were used to ascertain the symptoms present in the patients. Patients with checking symptoms, including dimensions of symptoms other than washing, were included in the checking subgroup. Patients with washing symptoms, including dimensions of symptoms other than checking, were included in the washing subgroup. If a patient had symptoms of cleaning/checking and other dimensions, we differentiated only one major symptom according to the OCI-R scores of each dimension [40]. For example, in the checking subgroup, checking symptoms had to be the most serious problem for the patient, and other dimensions of symptoms could not be scored higher than the checking dimension. In addition, patients with the following conditions were excluded from this study: (1) both doubt/aggression/checking and contamination/washing symptoms and (2) other dimensional symptoms that were considered to be the most serious problems. The OCD subtypes were finally identified by two experienced psychiatrists. Ultimately, 30 patients were included in the checking subgroup, and 15 patients were included in the washing subgroup.

### 2.4. fMRI Scanning

The MRI data were acquired using a Siemens 3.0 T scanner at the Department of Radiology, Nanjing Medical University Affiliated Nanjing Brain Hospital. All participants were in the supine position with their eyes closed in an awake, relaxed, and calm state during the scan. Moreover, the head was fixed with a sponge pad to reduce error caused by head movement, and earplugs were provided to reduce the perceived noise. The rs-fMRI data were acquired using an echo-planar imaging (EPI) sequence with the following parameters: repetition time (TR) = 2000 ms, echo time (TE) = 40 ms, field of view (FOV) = 240 × 240 mm, matrix = 64 × 64, flip angle (FA) = 90°, 36 slices, slice thickness = 4 mm, spacing between slices = 4 mm, and 240 volumes. T1-weighted anatomical images were obtained via the echo-planar imaging sequence (TR = 1900 ms, TE = 2.48 ms, FOV = 240 × 240 mm, matrix = 256 × 256, FA = 9°, 36 slices, slice thickness = 1 mm, spacing between slices = 0.5 mm, and 240 volumes).

### 2.5. MRI Data Preprocessing

All resting-state data were preprocessed using the Statistical Parametric Mapping (SPM12, http://www.fil.ion.ucl.ac.uk/spm, accessed on 11 March 2022) and RESTplus (http://restfmri.net/forum/restplus, accessed on 11 March 2022) software packages in the MATLAB toolbox. The above two software packages were added to “Set Path” in MATLAB 2013b. First, we sorted the raw data into T1, BOLD, and DWI by using REST DICOM Sorter in the RESTplus software. Then, we set the time points to “240” and TR to “2” in the RESTplus software and preprocessed the data as follows: (1) data format conversion from DICOM to NIFTI, (2) removal of the first 10 points, (3) slice timing, (4) realignment for head motion correction, (5) normalization by DARTEL using T1 image new segment, (6) smoothing, (7) detrending, (8) nuisance covariate regression of head motion signal, white matter signal, and cerebrospinal fluid signal, and (9) filtering. Participants with head motion exceeding 3 mm or 3° were excluded.

### 2.6. ReHo Analysis

The preprocessing process for the ReHo analysis included steps 1, 2, 3, 4, 5, 7, 8, and 9. Unsmoothed preprocessed data were used to calculate individual ReHo maps by the Kendall coefficient of harmony (KCC), which indicates the synchronization of the time series of the measured voxel with those of its neighboring 26 adjacent voxels. Subsequently, the individual KCC map of each voxel was divided by the whole-brain mean KCC for standardization, and finally, the SmKCCReHo maps obtained by spatial smoothing the standardized individual ReHo maps with a Gaussian kernel of 6-mm FWHM were used for the subsequent statistical analyses [28].

### 2.7. Seed-Based FC Calculation

The preprocessing process for the FC analysis included steps 1, 2, 3, 4, 5, 6, 7, 8, and 9. FC was analyzed by using a seed-based correlation analysis. Dysfunctional brain regions in the patients with the checking and washing subtypes of OCD were distinguished according to the results of the ReHo analysis, and abnormal regions > 20 voxels in the ANOVA were selected as regions of interest (ROIs). The FC map was obtained by a Pearson correlation analysis of the time series of the ROI with the time series of each brain voxel. Finally, the correlation coefficient “r” was transformed into Z values obeying a normal distribution using Fisher’s R-to-Z transformation [41].

### 2.8. Statistical Analysis

Regarding the demographic and clinical characteristics, the continuous variables were compared by a one-way ANOVA, and the categorical variables were compared by a chi-squared test. In addition, a two-sample *t* test was used to compare the OCD subgroups.

The statistical comparisons of ReHo and FC among the three groups were performed using a one-way ANOVA of the second level of a random effects analysis in SPM12. Subsequently, two-sample *t* tests were conducted between each pair of three groups to further identify the differences in each group. Age, sex, and education level were controlled as covariates in the ANOVA and post hoc test. Statistical inferences were made at the Gaussian random fields (GRF), voxel *p* < 0.001, and Cluster *p* < 0.05.

A receiver operating characteristic (ROC) curve was used to classify the ReHo values of different brain regions among the three groups. Brain regions with identification value were also included as ROIs for further FC analysis. In addition, we explored the relationship between ReHo and FC values with the clinical characteristics of OCD by using a correlation analysis.

## 3. Results

### 3.1. Demographic and Clinical Characteristics

Table 1 presents the demographic and clinical characteristics of the checking OCD, washing OCD, and HC groups. Age, sex, and education level did not significantly differ among the three groups. The Y-BOCS total, Y-BOCS compulsions, OCI-R washing, and OCI-R checking significantly differed between the patient groups. No significant differences between the checking and washing groups were found in the assessment of the other clinical characteristics.

The distribution of the symptom dimensions, comorbidities, and treatment history are shown in Table 2. In the checking group, 21 (70.0%) patients currently had other dimensional symptoms, including 12 (40.0%) patients with ordering symptoms, 1 (3.3%) patient with religious/sexual symptoms, one (3.3%) patient with hoarding symptoms, six (20.0%) patients with ordering and sexual/religious symptoms, and one (3.3%) patient with ordering, sexual/religious, and hoarding symptoms. In the washing group, five (33.3%) patients had other dimensional symptoms, including five (33.3%) patients with ordering symptoms and one (3.0%) patient with ordering and sexual/religious symptoms. Regarding the use of medication, in the checking group, 21 (70.0%) patients were previously or currently treated with medication, and 10 (33.3%) patients were drug-naïve. In the washing group, eight (53.3%) patients were previously or currently treated with medication, and seven (46.7%) patients were drug-naïve. Regarding the psychotherapy situation, 17 (56.7%) patients in the checking group had received psychotherapy, while none of the patients in the washing group had received psychotherapy. Finally, regarding comorbidities, nine (30.0%) patients had other psychiatric disorders in the checking group, including one (3.3%) patient with comorbid bipolar disorder (BP), one (3.3%) patient with comorbid BP, generalized anxiety disorder (GAD), and social anxiety disorder (SAD), three (10.0%) patients with comorbid MDD, one (3.0%) patient with comorbid MDD and SAD, and three (10.0%) patients with comorbid GAD, while only three (20.0%) patients with comorbid MDD were included in the washing group.

### 3.2. ReHo

**Figure 1:**
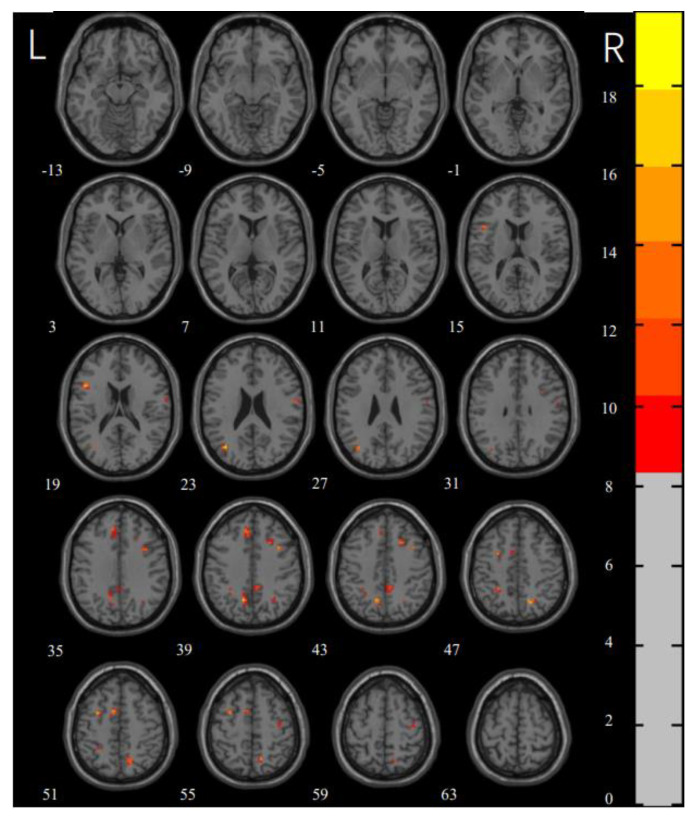
ANOVA differences in the ReHo values among the groups, washing group and healthy controls.

**Figure 2:**
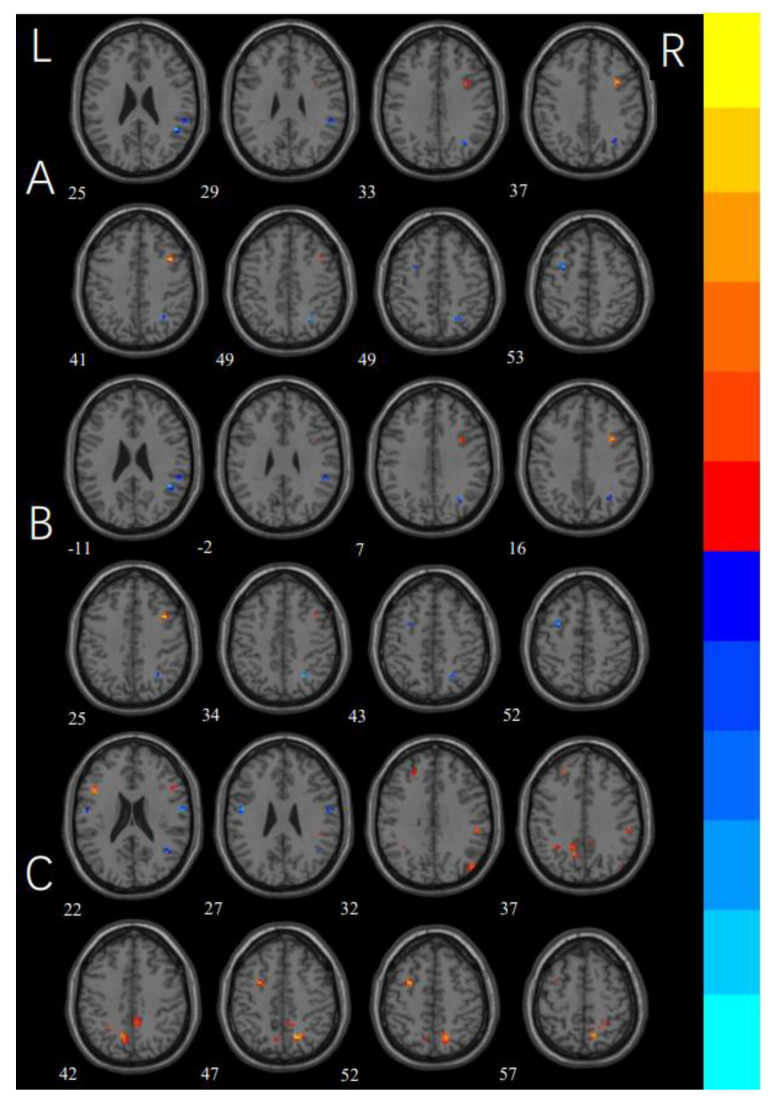
Post hoc comparisons of differences in the ReHo values among the checking group, washing group, and healthy controls. (**A**) Brain regions showing ReHo differences between the checking group and washing group. (**B**) Brain regions showing ReHo differences between the checking group and HCs. (**C**) Regions showing ReHo differences between the washing group and HCs. Warm colors show brain areas with increased ReHo values, while cool colors show decreased ReHo values.

The one-way ANOVA revealed significant differences in the ReHo maps among the three groups in the right superior frontal gyrus, left medial superior frontal gyrus, left inferior frontal gyrus, bilateral middle frontal gyrus, bilateral precuneus, left inferior parietal gyrus, left middle occipital gyrus, right precentral gyrus, right postcentral gyrus, and left supplementary motor area (Figure 1 and Table 3 for details). A post hoc test of three groups showed that compared with the HCs, the washing OCD group showed increased ReHo values in the left middle frontal gyrus, left inferior frontal gyrus, bilateral precuneus, right supramarginal gyrus, and left inferior parietal gyrus, while there were decreased values in the bilateral postcentral gyrus; the checking OCD group showed increased ReHo values in the right superior frontal gyrus, right medial orbital frontal gyrus, left medial superior frontal gyrus, bilateral precuneus, left angular gyrus, left inferior parietal gyrus, left middle occipital gyrus, left supplementary motor area, and the right Vermis, and decreased ReHo values in the bilateral middle temporal gyrus, left middle occipital gyrus, right precentral gyrus, and right postcentral gyrus. Compared with the washing OCD group, the checking OCD group showed increased ReHo values in the right middle frontal gyrus and decreased ReHo values in the left middle frontal gyrus, right supramarginal gyrus, right angular gyrus, and right inferior occipital gyrus (see Figure 2 and Table 4 for details).

### 3.3. ROC Curve Analysis

**Figure 3:**
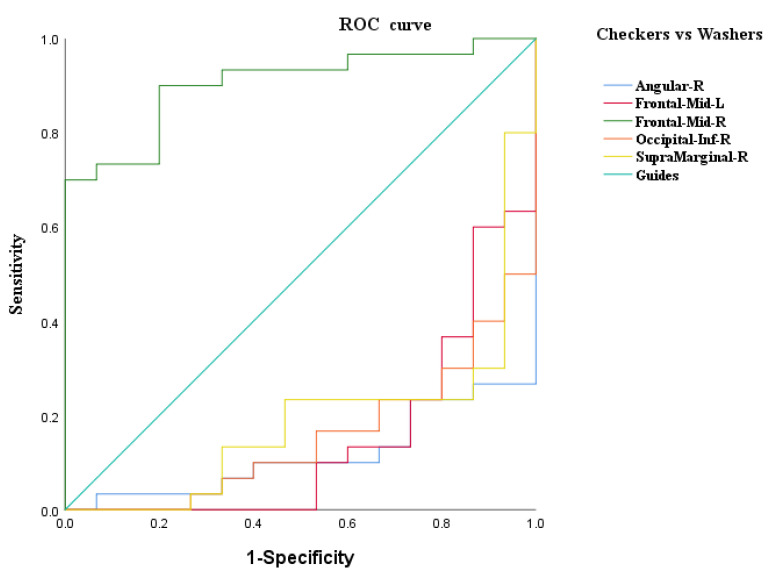
ROC curve analysis of ReHo differential brain regions between the checking and washing groups.

**Figure 4:**
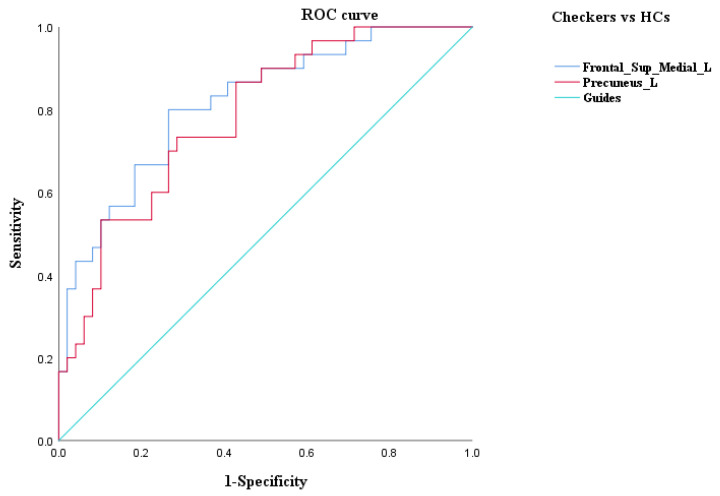
ROC curve analysis of ReHo differential brain regions between the checking group and HCs.

**Figure 5:**
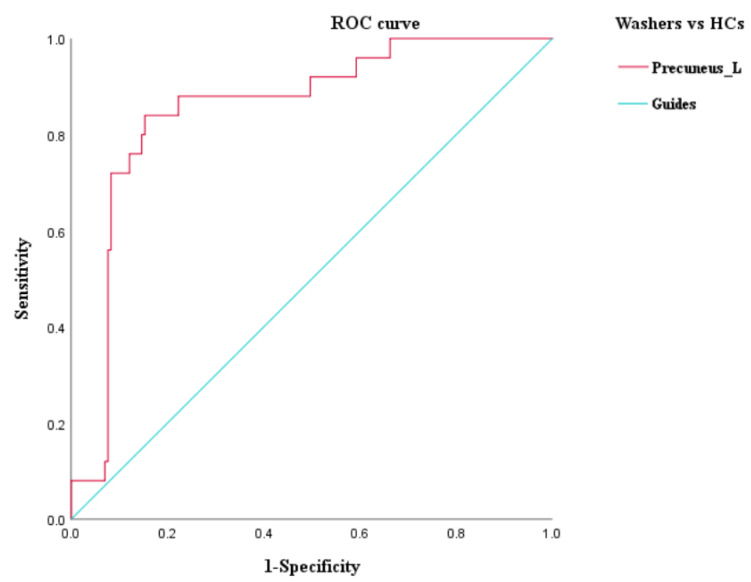
ROC curve analysis of ReHo differential brain regions between the checking group and HCs.

An ROC curve analysis was further performed to evaluate the identification value of ReHo of the ROIs obtained by ANOVA among the three groups and the brain regions that differed between the checking group and washing group. The results of the ROC curve analysis showed that (1) the ReHo values of the right middle frontal gyrus had a sensitivity of 90.0% and a specificity of 80.0% in the identification of checkers and washers; (2) the ReHo values of the left medial superior frontal gyrus had a sensitivity of 80.0% and a specificity of 80.0% in the identification of checkers and HCs; (3) the ReHo values of the left precuneus had a sensitivity of 73.3% and a specificity of 68.9% in the identification of checkers and HCs; and (4) the ReHo values of the left precuneus had a sensitivity of 80.0% and a specificity of 86.7% in the identification of washers and HCs (see Figure 3, Figure 4 and Figure 5, and Table 5 for details).

In summary, the ReHo values of the right middle frontal gyrus had a better discriminatory value for checking OCD and washing OCD. We also used the right middle frontal gyrus as ROI 3 for further FC analysis of the difference between checking and washing OCD. In addition, the ReHo values of the left medial superior frontal gyrus and left precuneus had a better discriminatory value for checking OCD and HCs, and the left precuneus had better discriminatory value for washing OCD and HCs; thus, the left precuneus may be important for distinguishing OCD patients from HCs.

### 3.4. Functional Connectivity

**Figure 6:**
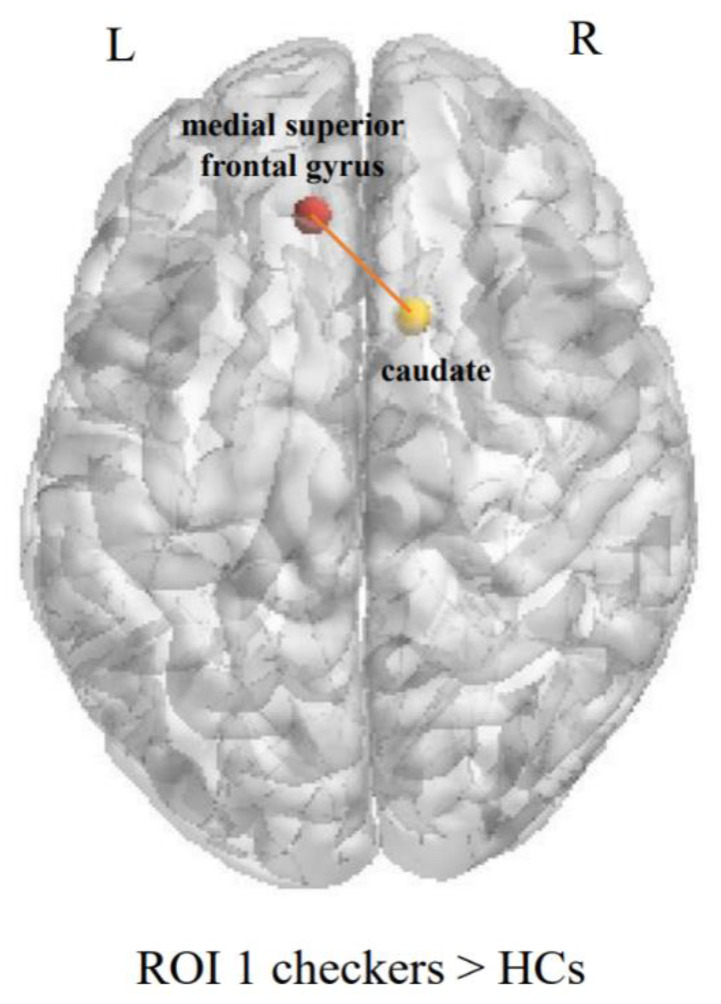
Brain regions showing functional connectivity differences between the checking and washing groups.

The ANOVA showed significant differences in the FC maps among the checking group, washing group, and HCs in the left medial superior frontal gyrus (ROI 1) and the right caudate. No differences in whole-brain functional connectivity were found in the left precuneus (ROI 2) among the three groups. A post hoc test of the three groups showed that the OCD patients showed higher FC between the left medial superior frontal gyrus and the right caudate than the HCs (see Figure 6 and Table 6 for details). In addition, based on the findings of the ROC curve analysis, the functional connectivity of the right middle frontal gyrus (ROI 3) showed differences between the checking and washing groups (see Table 6 for details).

### 3.5. Correlation Analysis

**Figure 7:**
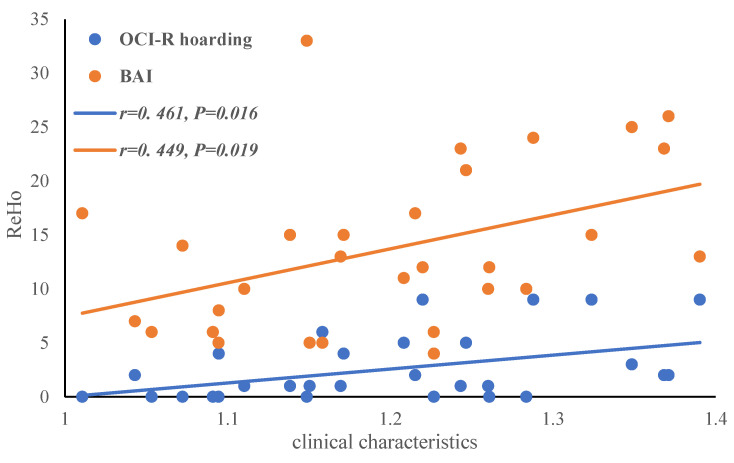
Bias correlation analysis between the left precuneus ReHo values and the OCI-R hoarding dimension and BAI.

In the checking group, the ReHo values of the left precuneus were positively correlated with the OCI-R hoarding dimension (*r* = 0.461, *p* = 0.016; uncorrected) and BAI (*r* = 0.449, *p* = 0.019; uncorrected). There were no significant correlations between the FC and clinical characteristics in the OCD subgroup (see Figure 7 and Table 7 for details).

## 4. Discussion

In this study, we compared spontaneous brain activity among the washing OCD, checking OCD, and HC groups based on resting-state fMRI techniques and further investigated symptom-specific brain network imaging markers by comparing the functional connectivity characteristics of the two types of patients from the perspective of functional brain networks. The results revealed extensive abnormalities in spontaneous brain activity involving frontal, temporal, and occipital regions in the patients compared to the HCs. The differences in local brain function between checking and washing OCD were mainly concentrated in the bilateral middle frontal gyrus, right supramarginal gyrus, right angular gyrus, and right inferior occipital gyrus. According to the ROC curve analysis, it was found that the right middle frontal gyrus had a better discriminatory value for checking and washing OCD, while the left precuneus had a greater value for distinguishing OCD patients from healthy subjects. Furthermore, the seed-based FC analysis revealed that the frontoparietal network was dysfunctional in OCD patients with checking.

Previous studies have supported dysfunction in the CSTC and extensive brain regions in OCD [41], which is consistent with the results obtained in the present study. We found that washing OCD patients had increased ReHo values in the left middle frontal gyrus, right supramarginal gyrus, right angular gyrus, and right inferior occipital gyrus, and decreased ReHo values in the right middle frontal gyrus compared to the checking OCD patients.

The middle frontal gyrus, an important component of the dorsolateral prefrontal cortex, is involved in many executive functions, such as planning, attentional regulation, cognitive flexibility, working memory, behavioral inhibition, and emotion regulation [42]. Studies have shown that spontaneous activity in the right superior frontal gyrus and middle frontal gyrus, which are involved in executive control, is diminished in washing OCD patients before they are ready to start cleaning behaviors [43]. This finding may be related to the fact that washing OCD inhibits the activity of core brain areas involved in executive control functions [43], which affects cognitive functions and enhances the urge to wash. In turn, the left middle frontal gyrus shows increased compensatory activity to maintain normal executive functions [44,45]. Therefore, dysfunction in the middle frontal gyrus may be a feature of washing OCD, and the right middle frontal gyrus in particular has a significant discriminatory value in distinguishing checking OCD from cleaning OCD.

The supramarginal gyrus and right angular gyrus form the inferior parietal lobule, which is the hub of the frontal–parietal network (FPN) and default-mode network (DMN) and plays an important role in bottom-up perception and social cognition [46]. The FPN, also known as the task-positive network, shows increased activity when attention is focused on an externally stimulating task, while the DMN shows diminished activity. The present study found that washing OCD showed stronger local activity in these brain regions involved in task activation than checking OCD. This finding may indicate that even in the resting state, washing OCD patients allocate more attention to the external environment, avoiding contact with possible external contamination, whereas checking OCD patients may allocate more attention internally to conduct processes, such as introspection and doubt. This finding is also consistent with the increased activation in the right angular gyrus in the checking OCD patients compared to the HCs in our study, which may reveal the unique symptomatic features and related neural mechanisms of the two types of OCD.

Also, in Y-BOCS scale results, we found that the total Y-BOCS score was higher in washers than checkers, but we consider that this may not indicate washers with more severe symptoms than checkers. As mentioned before, washing OCD patients allocated their attention to external stimulus, resulting in more overt compulsive behaviors that were more easily noticed by the patients and reported to the physicians, whereas the patients in the checking OCD patients may have indulged in introspection and pathological doubt, resulting in more mental ritual behaviors that were not easily noticed with the assessment of the Y-BOCS compulsion subscales, which suggests that the assessment of mental ritual behaviors should be not neglected for the OCD patients with checking symptoms.

The occipital cortex is involved in the pathophysiological mechanisms of OCD [47] and plays an important role in visual information processing, attention, and emotion processing [48,49]. The difference in ReHo in the right inferior occipital gyrus may indicate different degrees of visual processing impairment in the two different symptomatic subtypes of OCD. Our finding of abnormal spontaneous neural activity in the occipital gyrus supports the role of occipital cortex dysfunction in the pathophysiological mechanisms of OCD.

In addition, our study found that the left precuneus had a better discriminatory value for washing OCD patients and HCs and for checking OCD patients and HCs in the ROC curve analysis based on local functional indicators, suggesting that the left precuneus may be a potential imaging target for distinguishing OCD patients from HCs. The imaging results showed that a higher bilateral precuneus was found in both washing and checking OCD patients compared to the HCs. Anatomically, the precuneus is located in the medial part of the parietal cortex [50], which is a key hub of the DMN [51] that plays an important role in visuospatial memory, episodic memory retrieval, self-processing, consciousness, and higher-order cognitive functions [52,53,54]. In the resting state, patients with OCD exhibit enhanced functional connectivity of the DMN, which may be related to the patients’ hyperfocus on symptom-related thinking and potential external threats [55]. Additional structural imaging studies have noted that both OCD patients and their unaffected siblings show increased thickness in the right precuneus [51]. Higher precuneus reactivity is closely associated with OCD symptoms, whereas a structurally thickened cortex is consistent with functionally enhanced activity changes. The right precuneus may be a potential neural phenotype of OCD, whereas the left precuneus is of value in differentiating the OCD symptom subtypes; therefore, the precuneus is of great significance for the elucidation of the neural mechanisms of OCD.

Moreover, higher bilateral medial superior frontal gyrus reactivity was found in the OCD patients compared with the HCs. In particular, the left medial superior frontal gyrus has value in distinguishing OCD patients from HCs. The medial superior frontal gyrus is an important part of the medial prefrontal lobe, which is involved in memory, social, decision-making, emotional, and cognitive functions [56]. It has been suggested that the degree of enhanced spontaneous neural activity in the medial prefrontal lobe after fear extinction training may be correlated with the effect of fear memory extinction [57]. Reinforcement memory of safe experiences during fear extinction by stimulation of medial prefrontal activation serves as a useful adjunct to exposure therapy [58]. Therefore, the present study found enhanced activity in the bilateral medial superior frontal gyrus in checking OCD but not in washing OCD, which may predict the better efficacy of exposure with response prevention therapy in checking OCD.

Furthermore, the FC analysis revealed that checking OCD may involve frontal–striatal network dysfunction. Impairment in the frontal–striatal loop is a classic hypothesis of the neural mechanisms of OCD, which is further supported by studies of spontaneous functional brain network activity in checking OCD. Impaired autonomic awareness of situational memory, commonly referred to as memory deficits, is the core problem of checking OCD [59,60]. Furthermore, dysfunction in the frontal–striatal network may underlie the basis of the cognitive deficits in checking OCD [61]. When patients feel anxious, their decreased basal information processing capacity leads to more severe memory impairment, resulting in checking behaviors to reduce suspicion-induced anxiety. OCD patients with a high level of responsibility characteristics are more likely to have certain context-specific memory impairments [62]. Previous studies have reported that checking OCD patients tend to have a greater sense of responsibility [63]. In this paper, we found differences in functional connectivity between the checking and washing subtypes, which refer to cognitive deficits. Further studies are needed to explore the relationship between objective cognitive impairment and neural dysfunction.

### General Discussion of the Classification of the OCD Symptom Subtypes

Actually, the subtype of the OCD symptom dimension is sometimes ambiguous in the real world. Most OCD patients exhibit dynamic changes in obsession and compulsion in many different themes during the course of illness. For example, OCD patients with contamination/washing symptoms also show checking compulsion, which aims to ensure that they are not contaminated actually or mentally. This is still related to the core fear of contamination in washers as opposed to the pathological doubt of checkers. In addition, OCD patients with multiple dimensional symptoms will take a particular symptom as the main clinical manifestation [64]. Over time, the symptoms of adult OCD patients may change within specific symptom subtypes rather than across dimensions and tend to be stable [65]. Therefore, the clarification method of the OCD subtype of symptom dimension has been favored by many clinical researchers. The heterogeneity and complexity of OCD symptoms render the classification of the symptom subtypes uniquely significant. Understanding the psychological and neurological processes related to different subtypes of symptoms may be central to unraveling the etiology of OCD.

This study is deficient in the following aspects. First, a symptom-based classification differentiating the OCD subtype according to OCI-R and Y-BOCS may include mixed symptoms and affect the study results. Future research could classify precisely based on disease biomarkers using machine learning, such as structural neuroimaging biomarkers [66], inflammation-related biomarkers [67], and genetic and epigenetic architecture biomarkers [68,69]. Second, we did not exclude patients with comorbidities or medications. Thus, the impact of comorbidities or medications on the imaging findings could not be excluded. Third, we did not assess HCs with relevant scales of clinical symptom, leading to a lack of information concerning potential symptom dimensions in the normal population, and subsequent studies are necessary to fully assess healthy controls consistent with patients. Finally, the sample size in this study was relatively small, and future studies should conduct multicenter studies with larger sample sizes to improve the statistical validity.

In summary, based on the current data-driven analysis, we compared the spontaneous neural activity of brain regions and functional connectivity patterns of brain networks in washing and checking OCD using neuroimaging studies to reveal the pathophysiological mechanisms of different symptomatic subtypes of OCD. Notably, our findings suggest that there are extensive local differences in intrinsic spontaneous activity involving frontal, temporal, and occipital regions among the checking group, washing group, and HCs. Additionally, the differences in local brain function between checking and washing OCD were mainly concentrated in the bilateral middle frontal gyrus, right supramarginal gyrus, right angular gyrus, and right inferior occipital gyrus. The neural basis of checking OCD may be related to dysfunction in the frontal–striatal network, which distinguishes OCD from washing OCD.

## Figures and Tables

**Table 1:** Demographic and clinical characteristics of all participants.

Variable	Checkers	Washers	HCs	*F/χ*^2^/*t*	*p*
	(n = 30)	(n = 15)	(n = 45)		
Gender (male/female)	19/11	7/8	22/23	1.830	0.400 ^a^
Age (years)	28.57 ± 8.007	27.80 ± 7.702	25.91 ± 3.854	1.763	0.178 ^b^
Education (years)	15.67 ± 2.454	14.87 ± 2.924	15.89 ± 1.526	1.291	0.280 ^b^
Duration of illness > 5 years, n (%)	13 (43.3)	6 (40.0)	-	5.698	0.337 ^a^
Y-BOCS total	21.43 ± 5.876	25.40 ± 5.124	-	−2.223	**0.032** ^c^
Y-BOCS obsessions	11.27 ± 2.935	12.87 ± 2.875	-	−1.735	0.090 ^c^
Y-BOCS compulsions	10.17 ± 3.649	12.53 ± 2.416	-	−2.269	**0.028** ^c^
OCI-R total	23.47 ± 11.001	28.33 ± 8.304	-	−1.509	0.139 ^c^
OCI-R washing	2.57 ± 2.622	9.80 ± 2.111	-	−9.271	**0.000** ^c^
OCI-R hoarding	2.57 ± 3.081	2.27 ± 2.344	-	0.331	0.742 ^c^
OCI-R ordering	3.37 ± 3.102	3.07 ± 2.738	-	0.318	0.752 ^c^
OCI-R checking	5.50 ± 3.170	3.00 ± 2.619	-	2.634	**0.012** ^c^
BAI	13.70 ± 7.525	15.47 ± 11.728	-	−0.613	0.543 ^c^
BDI	14.53 ± 8.025	19.47 ± 12.065	-	−1.433	0.176 ^c^

Abbreviation: Y-BOCS, Yale–Brown Obsessive-Compulsive Scale; OCI-R, Obsessive Compulsive Inventory–Revised; BAI, Beck Anxiety Inventory; BDI, Beck Depression Inventory; ^a^ Pearson’s chi-squared test, ^b^ permutation ANOVA, ^c^ two-sample *t* test.

**Table 2:** Distribution of symptom dimensions, treatment history, and comorbidities in the patients.

Variable	Checkers		Washers	
Distribution of Symptom	Present (n = 21)	Past (n = 7)	Present (n = 6)	Past (n = 3)
washing	-	5	-	-
checking	-	-	-	3
ordering	19	4	6	-
sexual/religious	8	1	1	-
hoarding	2	-	-	-
Medication	n = 12	n = 13	n = 3	n = 6
	SSRIs (n = 11)	SSRIs (n = 15)	SSRIs (n = 3)	SSRIs (n = 6)
	SNRIs (n = 1)	SNRIs (n = 1)		
Psychotherapy	n = 16		n = 0	
Comorbidity	BP = 2, MDD = 4, GAD = 4, SAD = 4		MDD = 3	

Abbreviation: SSRIs, selective serotonin reuptake inhibiting antidepressants; SNRIs, serotonin and noradrenaline reuptake inhibiting antidepressants; BP, bipolar disorder; MDD, major depressive disorder; GAD, generalized anxiety disorder; SAD, social anxiety disorder.

**Table 3:** ANOVA differences in the ReHo values among the three groups.

	MNI x, y, z	Voxels	*F*	Side	Brain Region
CL1	−48, 15, 18	12	17.3371	L	inferior frontal gyrus
CL2	−36, −69, 24	14	19.83114	L	middle occipital gyrus
CL3	60, −6, 21	11	10.8797	R	postcentral gyrus
CL4	36, 12, 39	10	14.4576	R	middle frontal gyrus
CL5	18, −63, 48	17	16.4027	R	precuneus
CL6	−9, 36, 39	22	12.6563	L	medial superior frontal gyrus
CL7	−12, −60, 39	24	18.7764	L	precuneus
CL8	21, 18, 42	11	13.9385	R	superior frontal gyrus
CL9	−27, −45, 48	12	13.076	L	inferior parietal gyrus
CL10	−30, 3, 51	11	16.1396	L	middle frontal gyrus
CL11	−9, 6, 51	13	14.7148	L	supplementary motor area
CL12	39, −12, 57	11	11.5019	R	precentral gyrus

Abbreviations: CL, cluster; MNI, Montreal Neurological Institute space; L, left; R, right. The significance threshold was set at voxel *p* < 0.001 and Cluster *p* < 0.05 with GRF correction.

**Table 4:** Post hoc comparisons of differences in the ReHo values among the three groups.

	MNI x, y, z	Voxels	*T* Values	Side	Brain Region
Checker > Washers					
CL1	36, 12, 39	20	5.1965	R	middle frontal gyrus
Checker < Washers					
CL1	48, −81, −9	12	−4.1982	R	inferior occipital gyrus
CL2	54, −39, 27	13	−4.5237	R	supramarginal gyrus
CL3	24, −60, 45	11	−5.0666	R	angular gyrus
CL4	−30, 3, 51	15	−5.0536	L	middle frontal gyrus
Checkers > HCs					
CL1	0, −75, −24	16	4.7627	R	vermis
CL2	9, 66, −12	10	4.4357	R	medial orbital frontal gyrus
CL3	15, −48, 15	13	5.0067	R	precuneus
CL4	−39, −69, 24	19	5.8556	L	middle occipital gyrus
CL5	−9, 36, 39	41	4.9037	L	medial superior frontal gyrus
CL6	−42, −60, 36	13	4.2971	L	angular gyrus
CL7	−12, −60, 42	13	4.9284	L	precuneus
CL8	21, 18, 42	18	5.2585	R	superior frontal gyrus
CL9	−27, −45, 48	11	4.9786	L	inferior parietal gyrus
CL10	−9, 6, 51	33	5.1527	L	supplementary motor area
Checkers < HCs					
CL1	−48, −27, −3	12	−4.885	L	middle temporal gyrus
CL2	−48, −75, −6	19	−4.1681	L	middle occipital gyrus
CL3	45, −69, 6	15	−4.0421	R	middle temporal gyrus
CL4	51, −21, 54	17	−4.7041	R	postcentral gyrus
CL5	39, −12, 57	16	−4.4824	R	precentral gyrus
Washers > HCs					
CL1	−45, 15, 18	14	5.8462	L	inferior frontal gyrus
CL2	51, −30, 33	10	4.1435	R	supramarginal gyrus
CL3	−27, −51, 39	10	4.6492	L	inferior parietal gyrus
CL4	−12, −60, 39	39	5.1497	L	precuneus
CL5	6, −45, 42	25	4.1893	R	precuneus
CL6	18, −63, 48	23	5.6277	R	precuneus
CL7	−30, 3, 51	19	5.3576	L	middle frontal gyrus
Washers < HCs					
CL1	57, −6, 24	21	−4.2078	R	postcentral gyrus
CL2	−51, −6, 24	10	−4.2393	L	postcentral gyrus

Abbreviations: CL, cluster; MNI, Montreal Neurological Institute space; L, left; R, right. The significance threshold was set at voxel *p* < 0.001 and Cluster *p* < 0.05 with GRF correction.

**Table 5:** ROC curves of ReHo differential brain regions distinguished among the three groups.

Group	Brain Region	AUC	Demarcation	Sensitivity	Specificity
Checkers	Frontal-Mid-R	0.904	0.9140	90% (27/30)	80% (12/15)
Vs.	SupraMarginal-R	0.187	-	-	-
Washers	Occipital-Inf-R	0.153	-	-	-
	Frontal-Mid-L	0.147	-	-	-
	Angular-R	0.116	-	-	-
Checkers vs. HCs	Frontal_Sup_Medial_L	0.829	0.9768	80% (24/30)	80% (36/45)
	Precuneus_L	0.764	1.1370	73.3% (22/30)	68.9% (31/45)
Washers vs. HCs	Precuneus_L	0.849	1.1973	80% (24/30)	86.7% (39/45)

Abbreviations: Frontal-Mid-R, right middle frontal gyrus; SupraMarginal-R, right supramarginal gyrus; Occipital-Inf-R, right inferior occipital gyrus; Frontal-Mid-L, left middle frontal gyrus; Angular-R, right angular gyrus; Frontal_Sup_Medial_L, left medial superior frontal gyrus; Precuneus_L, left precuneus; AUC, area under the ROC curve.

**Table 6:** Brain regions showing functional connectivity differences based on the three ROIs among the checking group, washing group, and HC group.

Seeds	Group	MNI x, y, z	Voxels	*T* Values	Side	Brain Region
Frontal_Sup_Medial_L	Checkers > HCs	12, 15, 15	14	13.1601	R	caudate
Precuneus_L	-	-	-	-	-	-
Frontal_Mid_R	Checkers > Washers	48, −66, −48	4	3.9659	R	cerebelum_crus2
		−51, −36, −18	4	4.7955	L	inferior temporal gyrus
			2		L	middle temporal gyrus
		12, 66, −18	3	4.5508	R	superior orbital frontal gyrus
		−63, −48, −15	1	3.6373	L	inferior temporal gyrus
		−48, −3, 21	1	3.5563	L	precentral gyrus
		36, 12, 39	4	4.7987	R	middle frontal gyrus
		−12, 27, 42	1	3.5606	L	superior frontal gyrus
	Checkers < Washers	33, 33, −9	1	−3.6343	R	inferior orbital frontal gyrus

Abbreviations: Frontal_Sup_Medial_L, left medial superior frontal gyrus; Precuneus_L, left precuneus; Frontal_Mid_R, right middle frontal gyrus; MNI, Montreal Neurological Institute space; L, left; R, right. The significance threshold was set at voxel *p* < 0.001 and Cluster *p* < 0.05 with GRF correction.

**Table 7:** Analysis of the correlation between the ReHo and FC values with clinical scores in the checking and washing groups [r (*p*)].

		Y-BOCS **			OCI-R *						
ReHo	Brain Region	Total	Obsessions	Compulsions	Total	Washing	Hoarding	Ordering	Checking	BAI *	BDI *
Checkers	Left medial superior frontal gyrus	−0.172 (0.364)	0.035 (0.856)	−0.284 (0.128)	−0.059 (0.770)	−0.072 (0.720)	−0.267 (0.179)	0.023 (0.908)	−0.026 (0.898)	0.276 (0.163)	0.250 (0.208)
	Left precuneus	0.267 (0.154)	0.299 (0.108)	0.152 (0.423)	0.263 (0.185)	0.255 (0.199)	0.461 (0.016)	0.140 (0.486)	−0.106 (0.598)	0.449 (0.019)	0.176 (0.380)
Washers	Left medial superior frontal gyrus	−0.353 (0.197)	−0.161 (0.568)	−0.450 (0.092)	−0.006 (0.986)	−0.051 (0.876)	0.299 (0.344)	0.195 (0.544)	−0.287 (0.367)	0.200 (0.533)	0.312 (0.324)
	Left precuneus	−0.337 (0.220)	−0.220 (0.431)	−0.441 (0.100)	0.113 (0.727)	0.086 (0.791)	0.365 (0.244)	0.304 (0.337)	−0.229 (0.475)	0.200 (0.533)	−0.043 (0.894)
FC											
Checkers											
ROI 1-	Right caudate	−0.038 (0.842)	0.095 (0.616)	−0.051 (0.790)	−0.138 (0.492)	−0.206 (0.303)	−0.100 (0.621)	0.025 (0.900)	−0.092 (0.648)	−0.263 (0.184)	−0.028 (0.888)

Note: *, Pearson correlation analysis; **, Spearman correlation analysis. Abbreviation: ReHo, regional homogeneity, FC, functional connectivity; Y-BOCS, Yale–Brown Obsessive-Compulsive Scale; OCI-R, Obsessive-Compulsive Inventory–Revised; BAI, Beck Anxiety Inventory; BDI, Beck Depression Inventory; ROI 1, left medial superior frontal gyrus.

## Data Availability

Original data are available with the corresponding author N.L., but not archived in databases elsewhere.

## References

[1] Szalisznyó K., Silverstein D.N. (2021). Computational Predictions for OCD Pathophysiology and Treatment: A Review. Front. Psychiatry.

[2] Abramowitz J.S., Taylor S., McKay D. (2009). Obsessive-compulsive disorder. Lancet.

[3] Vellozo A.P., Fontenelle L.F., Torresan R.C., Shavitt R.G., Ferrão Y.A., Rosário M.C., Miguel E.C., Torres A.R. (2021). Symmetry Dimension in Obsessive–Compulsive Disorder: Prevalence, Severity and Clinical Correlates. J. Clin. Med..

[4] Zhang X., Liu J., Cui J., Liu C. (2013). Study of symptom dimensions and clinical characteristics in Chinese patients with OCD. J. Affect. Disord..

[5] Clair A.-H. (2020). Clinical characteristics of obsessivecompulsive disorder. La Revue du Praticien.

[6] Strauss A.Y., Fradkin I., McNally R.J., Linkovski O., Anholt G.E., Huppert J.D. (2019). Why check? A meta-analysis of checking in obsessive-compulsive disorder: Threat vs. distrust of senses. Clin. Psychol. Rev..

[7] Coleman S.L., Pieterefesa A.S., Holaway R.M., Coles M.E., Heimberg R.G. (2011). Content and correlates of checking related to symptoms of obsessive compulsive disorder and generalized anxiety disorder. J. Anxiety Disord..

[8] Pélissier M.-C., O’Connor K.P., Dupuis G. (2009). When doubting begins: Exploring inductive reasoning in obsessive-compulsive disorder. J. Behav. Ther. Exp. Psychiatry.

[9] Ron O., Oren E., Dar R. (2016). The doubt-certainty continuum in psychopathology, lay thinking, and science. J. Behav. Ther. Exp. Psychiatry.

[10] Toffolo M.B., Hout M.A.V.D., Engelhard I.M., Hooge I.T., Cath D.C. (2016). Patients With Obsessive-Compulsive Disorder Check Excessively in Response to Mild Uncertainty. Behav. Ther..

[11] Bhikram T., Abi-Jaoude E., Sandor P. (2017). OCD: Obsessive–compulsive … disgust? The role of. J. Psychiatry Neurosci..

[12] Brady R.E., Badour C.L., Arega E.A., Levy J.J., Adams T.G. (2021). Evaluating the mediating effects of perceived vulnerability to disease in the relation between disgust and contamination-based OCD. J. Anxiety Disord..

[13] Armstrong T., Olatunji B.O. (2017). Pavlovian disgust conditioning as a model for contamination-based OCD: Evidence from an analogue study. Behav. Res. Ther..

[14] Knowles K.A., Jessup S.C., Olatunji B.O. (2018). Disgust in Anxiety and Obsessive-Compulsive Disorders: Recent Findings and Future Directions. Curr. Psychiatry Rep..

[15] Mataix-Cols D., Cullen S., Lange K., Zelaya F., Andrew C., Amaro E., Brammer M.J., Williams S.C., Speckens A., Phillips M.L. (2003). Neural correlates of anxiety associated with obsessive-compulsive symptom dimensions in normal volunteers. Biol. Psychiatry.

[16] Murayama K., Nakao T., Sanematsu H., Okada K., Yoshiura T., Tomita M., Masuda Y., Isomura K., Nakagawa A., Kanba S. (2013). Differential neural network of checking versus washing symptoms in obsessive-compulsive disorder. Prog. Neuro-Psychopharmacol. Biol. Psychiatry.

[17] Via E., Cardoner N., Pujol J., Alonso P., López-Solà M., Real E., Contreras-Rodríguez O., Deus J., Segalàs C., Menchón J.M. (2014). Amygdala activation and symptom dimensions in obsessive–compulsive disorder. Br. J. Psychiatry.

[18] Mataix-Cols D., Wooderson S., Lawrence N., Brammer M.J., Speckens A., Phillips M.L. (2004). Distinct Neural Correlates of Washing, Checking, and Hoarding SymptomDimensions in Obsessive-compulsive Disorder. Arch. Gen. Psychiatry.

[19] Phillips M.L., Marks I.M., Senior C., Lythgoe D., O’Dwyer A.-M., Meehan O., Williams S.C.R., Brammer M.J., Bullmore E.T., McGuire P.K. (2000). A differential neural response in obsessive–compulsive disorder patients with washing compared with checking symptoms to disgust. Psychol. Med..

[20] Shapira N.A., Liu Y., He A.G., Bradley M.M., Lessig M.C., James G.A., Stein D.J., Lang P.J., Goodman W.K. (2003). Brain activation by disgust-inducing pictures in obsessive-compulsive disorder. Biol. Psychiatry.

[21] Thorsen A.L., Kvale G., Hansen B., Heuvel O.A.V.D. (2018). Symptom Dimensions in Obsessive-Compulsive Disorder as Predictors of Neurobiology and Treatment Response. Curr. Treat. Options Psychiatry.

[22] Agarwal S.M., Jose D., Baruah U., Shivakumar V., Kalmady S., Venkatasubramanian G., Mataix-Cols D., Reddy Y.C.J. (2013). Neurohemodynamic Correlates of Washing Symptoms in Obsessive-compulsive Disorder: A Pilot fMRI Study Using Symptom Provocation Paradigm. Indian J. Psychol. Med..

[23] Jhung K., Ku J., Kim S.J., Lee H., Kim K.R., An S.K., Kim S.I., Yoon K.-J., Lee E. (2014). Distinct functional connectivity of limbic network in the washing type obsessive–compulsive disorder. Prog. Neuro-Psychopharmacol. Biol. Psychiatry.

[24] Li P., Cheng J., Gu Q., Ruan H., Wang Y., Liu O., Wu Y., Wang Z. (2021). Influence of obsessive beliefs and impulsivity traits on symptom dimensions of obsessive-compulsive disorder patients. J. Shanghai Jiaotong Univ. (Med. Sci.).

[25] Yu J., Zhou P., Yuan S., Wu Y., Wang C., Zhang N., Li C.-S.R., Liu N. (2021). Symptom provocation in obsessive–compulsive disorder: A voxel-based meta-analysis and meta-analytic connectivity modeling. J. Psychiatr. Res..

[26] Ravindran A., Richter M., Jain T., Ravindran L., Rector N., Farb N. (2019). Functional connectivity in obsessive-compulsive disorder and its subtypes. Psychol. Med..

[27] Raimondo L., Oliveira A., Heij J., Priovoulos N., Kundu P., Leoni R.F., van der Zwaag W. (2021). Advances in resting state fMRI acquisitions for functional connectomics. NeuroImage.

[28] Ji L., Meda S.A., Tamminga C.A., Clementz B.A., Keshavan M.S., Sweeney J.A., Gershon E.S., Pearlson G.D. (2019). Characterizing functional regional homogeneity (ReHo) as a B-SNIP psychosis biomarker using traditional and machine learning approaches. Schizophr. Res..

[29] Shahhosseini Y., Miranda M.F. (2022). Functional Connectivity Methods and Their Applications in fMRI Data. Entropy.

[30] Hutchison R.M., Womelsdorf T., Allen E.A., Bandettini P.A., Calhoun V.D., Corbetta M., Della Penna S., Duyn J.H., Glover G.H., Gonzalez-Castillo J. (2013). Dynamic functional connectivity: Promise, issues, and interpretations. NeuroImage.

[31] Golestani A.M., Kwinta J.B., Khatamian Y.B., Chen J.J. (2017). The Effect of Low-Frequency Physiological Correction on the Reproducibility and Specificity of Resting-State fMRI Metrics: Functional Connectivity, ALFF, and ReHo. Front. Neurosci..

[32] Xu J., Guan X., Li H., Xu X., Zhang M. (2019). Integration and segregation of functional segmented anterior and posterior hippocampal networks in memory performance. Behav. Brain Res..

[33] Storch E.A., Rasmussen S.A., Price L.H., Larson M., Murphy T.K., Goodman W.K. (2010). Development and psychometric evaluation of the Yale–Brown Obsessive-Compulsive Scale—Second Edition. Psychol. Assess..

[34] Gong H., Nederpel T.M.H., Lin G., Zhang Y., Yang Y., Li B., Luo X., Fang F., Liu W., Zhang C. (2020). The Obsessive-Compulsive Inventory–Revised: Replication of the psychometric properties in China. Bull. Menn. Clin..

[35] Krámská L., Urgošík D., Liščák R., Hrešková L., Skopová J. (2020). Neuropsychological outcome in refractory obsessive–compulsive disorder treated with anterior capsulotomy including repeated surgery. Psychiatry Clin. Neurosci..

[36] Castro-Rodrigues P., Camacho M., Almeida S., Marinho M., Soares C., Barahona-Correa B., Oliveira-Maia A.J. (2018). Criterion Validity of the Yale-Brown Obsessive-Compulsive Scale Second Edition for Diagnosis of Obsessive-Compulsive Disorder in Adults. Front. Psychiatry.

[37] Hon S.K., Siu B.W., Cheng C.W., Wong W.C., Foa E.B. (2019). Validation of the Chinese Version of Obsessive-Compulsive Inventory-Revised. East Asian Arch. Psychiatry.

[38] Julian L.J. (2011). Measures of anxiety: State-Trait Anxiety Inventory (STAI), Beck Anxiety Inventory (BAI), and Hospital Anxiety and Depression Scale-Anxiety (HADS-A). Arthritis Care Res..

[39] Smarr K.L., Keefer A.L. (2011). Measures of depression and depressive symptoms: Beck Depression Inventory-II (BDI-II), Center for Epidemiologic Studies Depression Scale (CES-D), Geriatric Depression Scale (GDS), Hospital Anxiety and Depression Scale (HADS), and Patient Health Questionnaire-9 (PHQ-9). Arthritis Rheum..

[40] Hirose M., Hirano Y., Nemoto K., Sutoh C., Asano K., Miyata H., Matsumoto J., Nakazato M., Matsumoto K., Masuda Y. (2016). Relationship between symptom dimensions and brain morphology in obsessive-compulsive disorder. Brain Imaging Behav..

[41] Zhao Q., Xu T., Wang Y., Chen D., Liu Q., Yang Z., Wang Z. (2019). Limbic cortico-striato-thalamo-cortical functional connectivity in drug-naïve patients of obsessive-compulsive disorder. Psychol. Med..

[42] Del Casale A., Rapinesi C., Kotzalidis G.D., De Rossi P., Curto M., Janiri D., Criscuolo S., Alessi M.C., Ferri V.R., De Giorgi R. (2015). Executive functions in obsessive–compulsive disorder: An activation likelihood estimate meta-analysis of fMRI studies. World J. Biol. Psychiatry.

[43] Tang H., Lu X., Su R., Liang Z., Mai X., Liu C. (2017). Washing away your sins in the brain: Physical cleaning and priming of cleaning recruit different brain networks after moral threat. Soc. Cogn. Affect. Neurosci..

[44] Liu W., Liu L., Cheng X., Ge H., Hu G., Xue C., Qi W., Xu W., Chen S., Gao R. (2021). Functional Integrity of Executive Control Network Contributed to Retained Executive Abilities in Mild Cognitive Impairment. Front. Aging Neurosci..

[45] Jiang K., Xu Y., Li Y., Li L., Yang M., Xue P. (2022). How aerobic exercise improves executive function in ADHD children: A resting-state fMRI study. Int. J. Dev. Neurosci..

[46] Stern E.R., Fitzgerald K.D., Welsh R.C., Abelson J.L., Taylor S. (2012). Resting-State Functional Connectivity between Fronto-Parietal and Default Mode Networks in Obsessive-Compulsive Disorder. PLoS ONE.

[47] Fan J., Zhong M., Gan J., Liu W., Niu C., Liao H., Zhang H., Tan C., Yi J., Zhu X. (2017). Spontaneous neural activity in the right superior temporal gyrus and left middle temporal gyrus is associated with insight level in obsessive-compulsive disorder. J. Affect. Disord..

[48] Ljungberg M., Nilsson M.K.L., Melin K., Jönsson L., Carlsson A., Carlsson A., Forssell-Aronsson E., Ivarsson T., Carlsson M., Starck G. (2016). 1H magnetic resonance spectroscopy evidence for occipital involvement in treatment-naive paediatric obsessive–compulsive disorder. Acta Neuropsychiatr..

[49] Stern E.R., Muratore A.F., Taylor S.F., Abelson J.L., Hof P.R., Goodman W.K. (2016). Switching between internally and externally focused attention in obsessive-compulsive disorder: Abnormal visual cortex activation and connectivity. Psychiatry Res. Neuroimaging.

[50] Lee T.Y., Jung W.H., Bin Kwak Y., Yoon Y.B., Lee J., Kim M., Kim E., Kwon J.S. (2020). Distinct neural networks associated with obsession and delusion: A connectome-wide association study. Psychol. Med..

[51] Shaw P., Sharp W., Sudre G., Wharton A., Greenstein D., Raznahan A., Evans A., Chakravarty M.M., Lerch J.P., Rapoport J. (2014). Subcortical and cortical morphological anomalies as an endophenotype in obsessive-compulsive disorder. Mol. Psychiatry.

[52] Cavanna A.E., Trimble M.R. (2006). The precuneus: A review of its functional anatomy and behavioural correlates. Brain.

[53] Northoff G., Heinzel A., de Greck M., Bermpohl F., Dobrowolny H., Panksepp J. (2006). Self-referential processing in our brain—A meta-analysis of imaging studies on the self. NeuroImage.

[54] Watanabe T., Takezawa M., Nakawake Y., Kunimatsu A., Yamasue H., Nakamura M., Miyashita Y., Masuda N. (2014). Two distinct neural mechanisms underlying indirect reciprocity. Proc. Natl. Acad. Sci. USA.

[55] Beucke J.C., Sepulcre J., Eldaief M.C., Sebold M., Kathmann N., Kaufmann C. (2014). Default mode network subsystem alterations in obsessive–compulsive disorder. Br. J. Psychiatry.

[56] Lieberman M.D., Straccia M.A., Meyer M.L., Du M., Tan K.M. (2019). Social, self, (situational), and affective processes in medial prefrontal cortex (MPFC): Causal, multivariate, and reverse inference evidence. Neurosci. Biobehav. Rev..

[57] Cho J.-H., Deisseroth K., Bolshakov V.Y. (2013). Synaptic Encoding of Fear Extinction in mPFC-amygdala Circuits. Neuron.

[58] Quirk G.J., Garcia R., González-Lima F. (2006). Prefrontal Mechanisms in Extinction of Conditioned Fear. Biol. Psychiatry.

[59] Kalenzaga S., Clarys D., Jaafari N. (2020). The memory deficit hypothesis of compulsive checking in OCD: What are we really talking about? A narrative review. Memory.

[60] Alcolado G., Radomsky A.S. (2011). Believe in yourself: Manipulating beliefs about memory causes checking. Behav. Res. Ther..

[61] Tallis F. (1997). The neuropsychology of obsessive-compulsive disorder: A review and consideration of clinical implications. Br. J. Clin. Psychol..

[62] Moritz S., Jaeger A. (2017). Decreased memory confidence in obsessive–compulsive disorder for scenarios high and low on responsibility: Is low still too high?. Eur. Arch. Psychiatry Clin. Neurosci..

[63] Radomsky A.S., Alcolado G.M., Dugas M.J., Lavoie S.L. (2022). Responsibility, probability, and severity of harm: An experimental investigation of cognitive factors associated with checking-related OCD. Behav. Res. Ther..

[64] Starcevic V., Brakoulias V. (2008). Symptom Subtypes of Obsessive–Compulsive Disorder: Are they Relevant for Treatment?. Aust. N. Z. J. Psychiatry.

[65] Cervin M., Miguel E.C., Güler A.S., Ferrão Y.A., Erdoğdu A.B., Lazaro L., Gökçe S., Geller D.A., Yulaf Y., Başgül S. (2021). Towards a definitive symptom structure of obsessive−compulsive disorder: A factor and network analysis of 87 distinct symptoms in 1366 individuals. Psychol. Med..

[66] Bruin W.B., Taylor L., Thomas R.M., Shock J.P., Zhutovsky P., Abe Y., Alonso P., Ameis S.H., Anticevic A., Arnold P.D. (2020). Structural neuroimaging biomarkers for obsessive-compulsive disorder in the ENIGMA-OCD consortium: Medication matters. Transl. Psychiatry.

[67] Yuan N., Chen Y., Xia Y., Dai J., Liu C. (2019). Inflammation-related biomarkers in major psychiatric disorders: A cross-disorder assessment of reproducibility and specificity in 43 meta-analyses. Transl. Psychiatry.

[68] Bellia F., Vismara M., Annunzi E., Cifani C., Benatti B., Dell’Osso B., D’Addario C. (2020). Genetic and epigenetic architecture of Obsessive-Compulsive Disorder: In search of possible diagnostic and prognostic biomarkers. J. Psychiatr. Res..

[69] Bey K., Campos-Martin R., Klawohn J., Reuter B., Grützmann R., Riesel A., Wagner M., Ramirez A., Kathmann N. (2021). Hypermethylation of the oxytocin receptor gene (OXTR) in obsessive-compulsive disorder: Further evidence for a biomarker of disease and treatment response. Epigenetics.

